# Single and Multiple Infestation by Coccoid Pests in Coffee Shape Plant Chemical Defenses and Interactions Across Trophic Levels

**DOI:** 10.1021/acs.jafc.6c04549

**Published:** 2026-08-08

**Authors:** Enggel Beatriz Silva Carmo, Renato C. Macedo-Rego, Alexander N. Borg, John C. Caulfield, Bruna Corrêa-Silva, Axel Mithöfer, Arodí P. Favaris, José Mauricio Simões Bento, Maria Fernanda Gomes Villalba Peñaflor

**Affiliations:** † Departamento de Entomologia, 67739 Universidade Federal de Lavras (UFLA), Lavras 37203-202, Brazil; ‡ Translating Biotic Interactions, 15552 Rothamsted Research, Harpenden AL5 2JQ, U.K.; § LAGE do Departamento de Ecologia, Instituto de Biociências, 28133 Universidade de São Paulo, São Paulo 05508-090, Brazil; ∥ Research Group Plant Defense Physiology, Max Planck Institute for Chemical Ecology, Jena D-07745, Germany; ⊥ Departamento de Entomologia e Acarologia, Escola Superior de Agricultura Luiz de Queiroz, Universidade de São Paulo, Piracicaba 13418-900, Brazil

**Keywords:** biological pest control, induced plant defenses, multitrophic interactions, secondary plant metabolites, interspecific competition

## Abstract

Crop plants are attacked by multiple pests, inducing metabolic changes that shape interactions across trophic levels and influence the effectiveness of biological control. We examined how infestation by the coccoids *Coccus viridis* (scales) and *Planococcus citri* (mealybugs) modulates phytohormones, secondary metabolites, herbivore infestation, and behavior of the predator *Cryptolaemus montrouzieri* in Coffea arabica plants. Infestation by either scales or mealybugs activated the salicylate pathway but differentially regulated jasmonates and selected secondary metabolites. Scale infestation reduced mealybug performance, coinciding with elevated theophylline levels, whereas mealybugs activated jasmonates and upregulated several metabolites, including caffeine and catechin. The ladybug consumed coccoid pests and preferred mealybugs, but did not discriminate between scale- or mealybug-infested plant volatiles, suggesting limited specificity of herbivore-induced volatile blends in guiding predator foraging. Multiple infestation disrupted volatile-mediated attraction of the female ladybug. Our findings reveal the complexity of plant responses under multiple infestation and highlight species-specific interactions shaping tritrophic dynamics.

## Graphical abstract

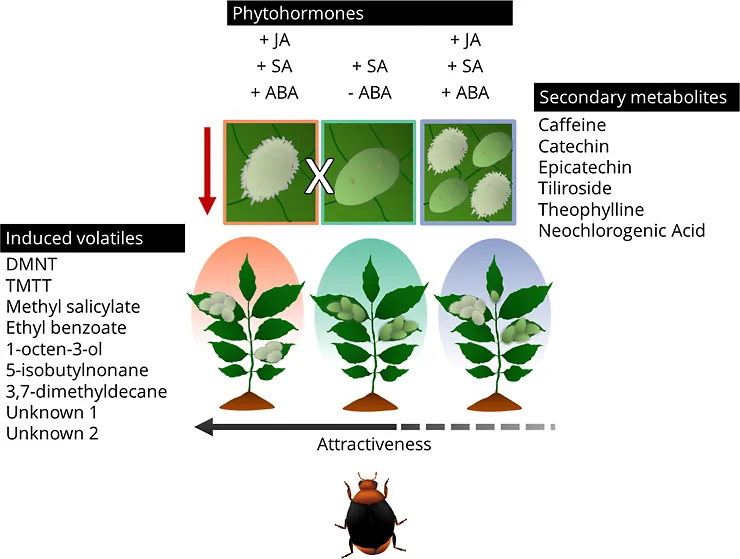

## Introduction

Plants are commonly attacked by multiple insect herbivores and must defend themselves to survive. To cope with these attackers, they have evolved constitutive and induced defense mechanisms that confer resistance against herbivores. Both constitutive and induced defenses rely on the synthesis of secondary metabolites, which function as regulators of growth and development or are induced in response to herbivore attack.^1^ When attacked, plants can produce toxic, deterrent or antinutritive compounds that directly affect the physiology and/or behavior of insect herbivores.^2,3^ In addition, herbivore attack can trigger the emission of a distinct blend of volatile organic compounds (VOCs), known as herbivore-induced plant volatiles (HIPVs), which attract natural enemies of the herbivores.^4,5^

Plants recognize herbivore attack or the imminence of herbivory through herbivore-associated cues, such as compounds in herbivore saliva, frass, honeydew, oviposition secretions and volatiles emitted by neighboring plants.^6,7^ Herbivory activates specific metabolic pathways responsible for the synthesis of defense compounds.^8,9^ The production of herbivore-induced plant defenses is mainly regulated by the orchestration of phytohormones, such as jasmonic acid (JA), salicylic acid (SA), abscisic acid (ABA), and ethylene. In particular, activation of the JA signaling pathway plays a crucial role in enhancing defenses against herbivores in general. These hormonal signaling pathways appear to be modulated according to the herbivore’s feeding mode, ensuring a tailored response to the attacking herbivore.^10,11^ For example, damage inflicted by chewing insects typically activates the jasmonate signaling pathway,^8,12,13^ whereas phloem-feeding insects predominantly trigger the salicylate pathway.^6,13,14^ These two pathways can act antagonistically: the accumulation of SA may inhibit JA signaling, rendering plants more susceptible to insect attack.^13,15^ This negative cross-talk between SA and JA has been identified as a strategy employed by phloem-feeding insects, such as aphids and mealybugs, to suppress JA-dependent plant defenses,^16,17^ thereby facilitating their feeding and establishment. Consequently, such suppression can favor the colonization by herbivores from different guilds (e.g., chewers) in a density-dependent manner.^18–21^

In natural and agricultural systems, plants often experience coinfestation by different herbivores, leading to complex ecological interactions. Co-occurring herbivores may avoid competition through temporal displacement or by altering their spatial distribution on the plant.^22–25^ The outcomes of these interactions seem to depend on factors such as herbivore feeding guilds, order of arrival, and herbivore density.^18,26–28^ However, while dominant herbivores may improve their own performance by suppressing plant defenses, this suppression can constrain the performance of subsequent herbivores.^28–31^ Asymmetrical facilitation has been observed among phloem-feeding insects sharing the same plant, whereby one species negatively affects the performance of a later-arriving species.^17,28–30,32^ Co-infestation by multiple phloem-feeding insects can induce distinct chemical defense profiles in plants, including changes in production of phytohormones, secondary metabolites, and VOCs.^29,30,33^ These herbivore-driven dynamics influence plant-mediated cascade responses, including HIPVs and their attractiveness to natural enemies of the herbivores, potentially affecting their foraging efficiency in the presence of herbivores from different guilds or nonprey/nonhost species.^34–37^

Natural enemies associate specific HIPVs with the presence of their prey or hosts, using these cues to enhance their foraging efficiency.^38,39^ The profile of HIPVs varies depending on herbivore identity, as well as plant species, age, and physiological state.^1,5,40,41^ This variation results in the emission of qualitatively distinct volatile blends and differences in the levels of phytohormone activation.^41,42^ The presence of multiple herbivores can modify the composition of HIPV blends,^22,43–45^ and the presence of a nonprey (or nonhost) may negatively affect the foraging of some natural enemies to locate their prey or host.^34,36,37^ However, natural enemies do not necessarily lose their ability to locate prey and hosts on multiple-infested plants. Instead, they remain attracted to the HIPVs associated with the presence of their specific host or prey 46. This suggests that chemical communication remains stable through conserved search-template composition, despite the complexity of herbivore communities.^39,46,47^ Conversely, the presence of multiple prey species does not necessarily result in enhanced attraction of natural enemies.^44,46,48,49^

Coffee (*Coffea arabica* L.) (*Rubiaceae*), a crop of major economic importance, is chemically rich and contains a diverse array of secondary metabolites, including alkaloids, flavonoids, terpenoids, xanthones and phenolic compounds.^50–52^ Coffee plants are susceptible to infestation by various insect herbivores that are considered pests in the agronomic context. Among phloem-feeding insects, members of the superfamily Coccoidea, such as the citrus mealybug *Planococcus citri* (Risso) (Hemiptera: Pseudococcidae) and the green scale *Coccus viridis* (Green) (Hemiptera: Coccidae), are particularly important pests with overlapping distributions in Brazil.^53–55^ These insects cause both direct and indirect damage, including stunting, virus transmission, defoliation, and reduced photosynthetic efficiency.^56,57^ Dispersal to suitable feeding sites on the plant occurs primarily during the early, more mobile nymphal stages; once settled, females exhibit a sedentary lifestyle during their adult stage.^56,58,59^

*P. citri* additionally produces a protective wax layer that covers its body and ovisacs.^60^ Similar to other hemipterans, this mealybug employs detoxifying enzymes in its saliva and harbors symbiotic microorganisms that facilitate feeding.^61–63^ Previous studies have shown that infestation by *P. citri* induces accumulation of both JA and SA in coffee plants, rendering them more resistant to subsequent attack by the red spider mite *Oligonychus ilicis* (McGregor) (Acari: Tetranychidae).^34^ Moreover, the sedentary behavior of these phloem-feeding insects, combined with continuous feeding, leads to honeydew accumulation on leaves, which promotes the development of sooty mold.^64^ Honeydew deposits by itself can induce plant defense responses, acting as chemical signals from the host, and exhibiting chemical properties that serve as host-associated cues for natural enemies.^35,65,66^

In the case of *Co. viridis*, honeydew accumulation promotes the growth of sooty mold, forming black superficial colonies that significantly reduce photosynthetic activity.^67^ Green scales have been reported to inject toxins into vascular tissues, leading to phloem cell rupture and pronounced alterations in coffee plant metabolism.^53,56,68^ Interestingly, certain coffee plant compounds, such as chlorogenic acid and caffeine, stimulate the locomotion of green scales without significantly increasing mortality, potentially enhancing their dispersal.^69,70^

*Cryptolaemus montrouzieri* Mulsant (Coleoptera: Coccinellidae) is a coccidophagous predator widely introduced for the biological control of mealybugs, with a broad prey range.^71–73^ This ladybug has demonstrated the ability to detect plant volatiles associated with prey infestation^74^ and to discriminate between plants infested by its prey and those infested by nonprey species.^34^ The presence of nonprey herbivores, such as mites, on plants coinfested with mealybugs results in a distinct repellent effect on *Cr. montrouzieri*. The ladybug exhibits a clear aversion to a volatile blend emitted in the presence of nonprey arthropods.^34^ This indicates that herbivory by multiple species can disrupt the attraction of *Cr. montrouzieri*, hindering its ability to effectively locate mealybugs, ultimately reducing its efficiency in controlling mealybug populations.

Here, we investigated how herbivory by *P. citri* and *Co. viridis* on coffee plants alters plant chemical defenses and its subsequent effect on herbivore and chemical mediated tritrophic interactions with the biological control agent *Cr. montrouzieri*. Based on the evidence that phloem-feeding insects predominantly induce SA-mediated defenses and may suppress or weakly induce JA-mediated responses through negative cross-talk,^13,14^ we addressed five key hypotheses related to these interactions: (1) herbivory by phloem-feeding insects facilitates the colonization and establishment of a subsequent nonconspecific phloem-feeding insect by increasing the salicylate signaling and suppressing the jasmonate signaling; (2) single infestations by phloem-feeding insects results in species-specific regulation of herbivore-induced plant defenses; (3) multiple infestations by both phloem-feeding species has nonadditive effects (synergistic or antagonistic) effects on herbivore-induced plant defenses compared with single infestations on coffee plants; (4) the predator prefers volatile blends emitted by plants infested with its preferred prey; and (5) the predator is equally attracted to volatiles from plants infested by two phloem-feeding herbivores compared to plants infested by a single prey. To test these hypotheses, we conducted a series of behavioral assays and chemical analyses to characterize direct and indirect herbivore-induced plant defenses and their hormonal regulation. Overall, our study helps to understand how infestation by phloem-feeding pests of the superfamily Coccoidea shapes subsequent interactions across trophic levels, which are important for predictions of pest dynamics in the field and the development of effective and sustainable pest management strategies.

## Materials and Methods

### Plants and Insects

Seeds of *C. arabica* cv. Mundo Novo were planted in polyethylene bags (20 cm × 10 cm) filled with a mixture of soil, commercial substrate (Tropstrato HA, Vida Verde, Mogi Mirim, Brazil), and sand (2:1:1, v/v). Plants were grown in a greenhouse (Lavras, MG, Brazil, 21°13′35.1″ S, 44°58′30.6″ W), under natural temperature and light fluctuations, from July 2021 through January 2022 and from June 2022 to December 2023. All bioassays were conducted using coffee plants aged between 7 and 9 months. Founding colonies of the mealybug *P. citri* were collected from cocoa plants (*Theobroma cacao* L.) (Lavras, MG, Brazil) and maintained in the laboratory on pumpkin fruits (*Cucurbita maxima* L. cv. Cabotchá), previously washed with neutral soap and sodium hypochlorite, and kept in cages. The scale *Co. viridis* was collected from coffee leaves in Lavras, Brazil. The insects were transferred by contact, using previously infested leaves, to greenhouse coffee plants protected by cages covered with voile fabric to avoid contamination by other insects. The colony of the ladybug *Cr. montrouzieri* was established with ∼500 individuals obtained from the rearing facility at Embrapa Mandioca e Fruticultura (Cruz das Almas, Brazil). Ladybug larvae and adults were maintained in plastic cages with side openings covered with fine-mesh fabric for ventilation and fed on *P. citri* reared on pumpkins infested in the laboratory for one month. All insect colonies were maintained in a room under controlled conditions (25 ± 2 °C, 70 ± 10% RH, 12 h light: 12 h dark).

### Plant Treatments

Mealybug-infested plants were obtained by transferring 50 mealybugs (nymphs and adults) collected from pumpkins in the rearing colony to a midthird leaf pair of coffee plants.^75^ Unlike mealybugs, scales are much less mobile and typically keep their stylets inserted into the leaf unless the tissue dries out.^69^ To obtain scale-infested plants, a leaf from rearing plants containing about 50 scales (nymphs and adults) was clipped onto a midthird leaf pair of coffee plants. Since neither mealybugs nor scales exhibited a preference for plants already colonized by the other species (see results for host preference by phloem-feeding insect assays), multiple-infested coffee plants were obtained by simultaneously infesting plants with both species. For each species, the same number of individuals and methods as in the single-infestation treatments. Thus, multiple-infested plants received twice the total number of insects compared with single-infested treatments (Figure 1A). To prevent insect escape, each infested plant was individually bagged with fine-mesh fabric. All plants (single and multiple infestations) were maintained for a total of 30 days after the initial infestation before being used in the experiments. Uninfested plants were also bagged but kept insect-free in the same room as infested treatments for the same duration.

**Figure 1:**
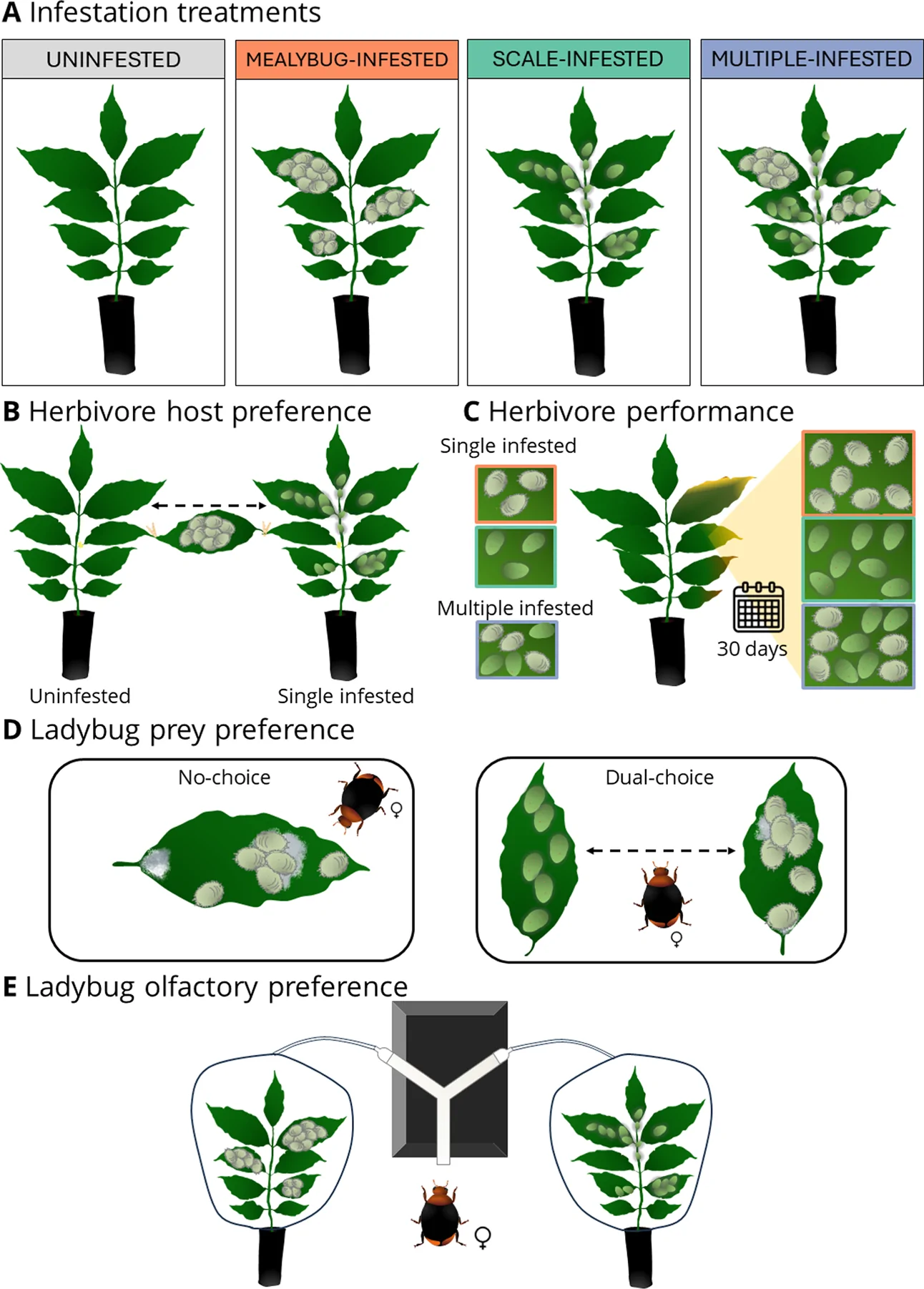
(A) Illustrative summary of coffee (*Coffea arabica*) plant treatments: uninfested (control), mealybug-infested (*Planococcus citri*), scale-infested (*Coccus viridis*), and multiple-infested plants (scale + mealybug); (B) phloem-feeding insect preference dual-choice assay; (C) phloem-feeding insect performance on single infested and multiple-infested plants; (D) female ladybug (*Cryptolaemus montrouzieri*) prey preference in a nonchoice arena assay and a dual-choice; and (E) ladybug olfactory preference in a Y-tube olfactometer assay between different treatments of infestation.

### Host Preference by Phloem-Feeding Insects

To evaluate whether herbivory by nonconspecific phloem-feeding insects hinders or facilitates colonization by the other species, a two-choice assay was conducted to assess the preference of mealybugs and scales for uninfested and infested coffee plants (Figure 1B). Without excising it from the plant, a single leaf from the midthird portion of each plant was isolated at the petiole using a 1:1 mixture of entomological glue and lanolin, thereby preventing the tested insects from spreading across the plant. The isolated leaves of the plant pair were placed in contact, providing the point from which insects could choose between them. To assess mealybug host preference, a detached coffee leaf infested with approximately 50 mealybugs (nymphs and adults) was clipped onto the isolated leaf of both an uninfested coffee plant and a scale-infested plant, positioned on opposite sides. This assay was conducted with 20 replicates. The scale host preference for uninfested and mealybug-infested coffee plants was assessed using a similar experimental setup, but the detached coffee leaf carrying approximately 50 scales (nymphs and adults) was clipped onto the leaf of each plant. This assay was conducted with 25 replicates. This experiment was carried out in a greenhouse under natural oscillations of temperature, light, and humidity (March to May, 2023; Lavras, Brazil). For each replicate, the position of plant pairs was randomized within the greenhouse benches, and plant positions were rotated every 3 days to minimize potential spatial effects. The number of insects moving to each plant was recorded by counting the individuals present on each plant after 3, 6, and 9 days.

### Phloem-Feeding Insects’ Performance

The performance of phloem-feeding insects, under single and multiple infestation treatments, was assessed by counting the number of individuals on the plants 30 days after infestation. The midthird leaf pair of uninfested coffee plants was infested using detached coffee leaves carrying either 50 mealybugs (nymphs and adults) or 50 scales (nymphs and adults), following the release method described in the host preference assay. To assess the performance of both phloem-feeding insects in multiple infestations, coffee plants were simultaneously infested with the same numbers used in the single-infestation treatments (Figure 1C). The plants were then enclosed in fine-mesh fabric bags to prevent insect escape. After 30 days, the numbers of nymphs and adults of each species present on the entire plant were recorded by counting all individuals across all leaves and stems. Twelve replicates were conducted in the greenhouse under natural oscillations of temperature, light and humidity (March to May, 2023; Lavras, Brazil).

### Phytohormones and Target Analysis of Secondary Metabolites

The fourth leaf pair from uninfested, mealybug-infested, scale-infested and multiple-infested coffee plants was collected 30 days after infestation. The leaves were gently cleaned with cotton moistened with distilled water to remove adhering insects and immediately frozen in liquid nitrogen. Samples were lyophilized for 27 h at −45 °C in a freeze-dryer (L101, LIOTOP, LIOBRAS, São Carlos, Brazil). The dried leaves were ground into a fine powder and stored in microtubes. For the extraction of putative defensive secondary metabolites, 50 mg of freeze-dried leaf tissue was diluted in 500 μL of methanol (99.8%, Fisher Scientific, Loughborough, UK). The mixture was sonicated for 25 min to facilitate phytochemical extraction and then centrifuged at 30,000 rpm for 10 min to separate the solid residues from the liquid extract. The supernatant was filtered through glass fiber to remove remaining particulates. To standardize filtered volumes, samples were concentrated using a nitrogen concentrator (Blowdown Evaporator, Radleys, Saffron Walden, UK), followed by the addition of 500 μL of methanol and sonication for 25 min. The LC–MS analysis was performed using an Acquity ultraperformance liquid chromatography (UPLC) system coupled to a Synapt G2Si Q-Tof mass spectrometer with an electrospray ionization (ESI) source (Waters Corporation, Manchester, UK), operated using Masslynx 4.1 software. Chromatographic separation was performed using a C18 reverse-phase column (BEH C18 2.1 × 50 mm, 1.7 μm; Waters, Manchester, UK) at a flow rate of 0.21 mL. min^–1^. The column was maintained at 50 °C and the injection volume was 3 μL. The mobile phase consisted of solvent A (0.01% formic acid v/v, water) and solvent B (0.01% formic acid v/v in methanol), which were used at a flow rate of 0.21 mL·min^–1^, with the following gradient: 0–3 min, 95% A; 3–7 min, 95% A; 7–11 min, 85% A; 11–13 min 75% A; 13–18 min, 70% A; 18–25 min, 50% A; 25–30 min, 25% A; 30–39.1 min, 0% A; 39.1–43 min, 95% A, for a total time of 43 min. A caffeine solution (100 ng. μL^–1^) was used as a reference standard for signal normalization. Mass spectra of coffee metabolites were compared with available authentic standards and reference spectra reported in 51. When authentic standards were not available, compound annotation was performed based on accurate mass (*m*/*z*) and molecular formula information by comparison with entries from the PubChem database.^76^ Individual compounds were relatively quantified based on peak areas normalized to a caffeine reference and corrected for sample weight, as no compound-specific calibration curves were performed.

For the phytohormonal analyses, a 30 mg aliquot of freeze-dried leaf tissue was used. A total of 1.5 mL of methanol and 4 μL of internal standard (D6JA, D4SA, D6ABA: 10 ng. μL^–1^, and D6-JA-Ile: 2 ng. μL^–1^ in MeOH) were added to each sample, which was shaken for 30 min and centrifuged at 13,000 rpm at 4 °C for 20 min. The supernatant was transferred to new microtubes on ice. The pellet from the first extraction was resuspended in 500 μL of methanol, shaken, and centrifuged again. The resulting supernatant was combined with the first extract. The supernatant was concentrated by vacuum centrifugation. After concentration, 500 μL of methanol was added, and the mixture was vortexed and centrifuged at 16,000 rpm at 4 °C for 7 min. A 400 μL aliquot of the supernatant was collected for analysis. Phytohormone analysis was performed by LC–MS/MS as described in,^77^ using an Agilent 1260 series HPLC system (Agilent Technologies) coupled to a QTRAP 6500 tandem mass spectrometer (SCIEX, Darmstadt, Germany). Chromatographic separation was carried out on a Zorbax Eclipse XDB-C18 column (50 × 4.6 mm, 1.8 μm, Agilent Technologies), with water containing 0.05% formic acid (mobile phase A) and acetonitrile (mobile phase B). The elution profile was: 0–0.5 min, 10% B; 0.5–4.0 min, 10–90% B; 4.0–4.02 min, 90–100% B; 4.02–4.5 min, 100% B; 4.51–7.0 min, 10% B. The flow rate was 1.1 mL·min^–1^, and the column temperature was maintained at 25 °C. The mass spectrometer was operated in negative ionization mode with a Turbo Spray ion source. The ion spray voltage was −4500 eV, and the turbo gas temperature was set to 650 °C. Nebulizing gas was set at 60 psi, curtain gas at 40 psi, heating gas at 60 psi, and collision gas at ″medium″. Multiple reaction monitoring (MRM) mode was used. Since both D6JA and D6-JA-Ile standards contained 40% D5 compounds, the peak areas of both D5 and D6 compounds were summed for quantification.

### Prey Preference of *Cr. montrouzieri*

The predator preference for consuming mealybugs and scales was assessed using both nonchoice and dual-choice assays (Figure 1D). In the nonchoice assay, a single unmated adult female ladybug, previously starved for 48 h, was released at the center of a rectangular plastic arena (24 × 15 cm). Each arena contained an excised coffee leaf bearing 50 individuals (nymphs and adults) of either mealybugs or scales, positioned at the center of the arena. In the dual-choice assay, the setup was similar except that two coffee leaves, one bearing mealybugs and the other scales, were placed on opposite sides of the arena, allowing the *Cr. montrouzieri* female to choose between them from the arena center. Twenty replicates were conducted under controlled laboratory conditions (25 ± 2 °C, 70 ± 10% RH, 12 h light: 12 h dark). The position of the arenas on the laboratory benches was randomized, and the position of the coffee leaves (left/right) was randomized for each replicate to minimize potential spatial effects. The ladybugs were removed after 24 h, and prey consumption was estimated by subtracting the remaining mealybugs and scales from the initial numbers at the start of the assay. In the dual-choice assay, the proportion of each phloem-feeding insect consumed was estimated relative to the total number of insects provided.

### Olfactory Preference of *Cr. montrouzieri* to Plant Volatiles

The olfactory preference of *Cr. montrouzieri* females for volatiles emitted from uninfested and infested coffee plants was evaluated in Y-tube olfactometer bioassays, following the method described in.^34^ A glass Y-tube olfactometer with equal side arms (15 cm long and 4 cm in external diameter) was positioned vertically inside a cardboard box (Figure 1E). The vertical orientation was used to account for the ladybug’s negative geotropism^34^ whereas the box aimed at minimizing visual cues. Coffee plants were individually enclosed in polyester bags (41 cm × 33 cm) with openings at the top to connect the airflow tubes. To eliminate potential visual stimuli for the ladybugs, the bags enclosing the plants were covered with white paper. Charcoal-filtered and humidified air was drawn through the olfactometer system at a flow rate of 1.5 L·min^–1^ using an air pump.

Prior to assays, 10 day-old unmated adult female ladybugs were isolated from the laboratory colony and starved for 24 h. Each female was then released into the central arm of the Y-tube, and its choice was recorded when it crossed a line located in the distal third of one of the lateral arms (10 cm from the center) within a 5 min period. Each insect was tested only once. Individuals that did not make a choice within the 5 min, were considered “non-responsive″ and excluded from the analysis. To avoid directional bias, the position of the treatments (left/right) was switched after each trial. Additionally, to reduce bias from chemical cues left by the ladybugs on the surface of the glass, the Y-tube olfactometer was replaced with a clean unit after every five insects tested. Bioassays were performed under controlled laboratory conditions (25 ± 2 °C, 70 ± 10% RH). The olfactory preference of ladybugs was assessed for the following treatment combinations: (i) uninfested plant vs. mealybug-infested plant; (ii) uninfested plant vs. scale-infested plant; (iii) scale-infested plant vs. mealybug-infested plant; (iv) multiple-infested plant vs. mealybug-infested plant; (v) multiple-infested plant vs. scale-infested plant; and (vi) multiple-infested plant vs. uninfested plant. For each assay, six plant pairs were used, with ten adult female ladybugs tested per pair.

### Plant Volatiles

Volatile emissions from mealybug-infested, scale-infested, multiple-infested, and uninfested plants were collected using a push–pull aeration system for 8 h during the photophase (from 8:00 a.m. to 4:00 p.m.) in the laboratory. Plants were maintained under artificial light (UV LED Full Spectrum Grow Light, 2000 lx). Each plant was enclosed in polyester bags (41 cm × 33 cm) and connected to the aeration system with an incoming airflow of 0.5 L·min^–1^ filtered through activated charcoal and a triple filter (TRIO-02, ARFUSION, Americana, São Paulo, Brazil). Volatiles were captured using thin glass tubes filled with 30 mg of HayeSep-Q adsorbent polymer (80/100 mesh; Hayes Separation Inc., Bandera, TX, USA). Air was drawn through the system at 250 mL·min^–1^ using a vacuum pump (SKC Pocket Pump TOUCH, SKC Ltd., Blandford Forum, UK). The adsorbent filters were eluted with 100 μL of hexane (Sigma-Aldrich, St. Louis, USA). A 10 μL volume of a 20 ng·μL^–1^ solution of nonyl acetate was added to each sample as an internal standard. The extracts were stored in sealed glass vials in a −30 °C freezer.

A 2 μL aliquot of each extract was injected at 250 °C into a gas chromatograph with a flame ionization detector (GC-FID; Shimadzu GC-2010, Shimadzu Corp., Kyoto, Japan) equipped with an Rtx-1 column (30 m × 0.25 μm × 0.25 mm; Restek Corporation, Bellefonte, PA, USA) using helium as the carrier gas at a linear velocity of 25 cm·s^–1^. The column temperature was held at 40 °C for 5 min, then increased to 150 °C at 5 °C·min^–1^ and finally raised to 250 °C at 20 °C·min^–1^ and held for 30 min.

A 2 μL aliquot of each representative sample was also run in a gas chromatograph coupled to a mass spectrometer (GC–MS; GCQP-2010 Ultra, Shimadzu Corp., Kyoto, Japan) equipped with an Rtx-1MS column (30 m × 0.25 μm × 0.25 mm; Restek Corporation, Bellefonte, PA, USA), using a similar method to the GC-FID. The ion source and transfer line were maintained at 250 °C for electron impact analysis at 70 eV (35–300 *m*/*z*). Total volatile emission was estimated as the sum of all peak areas detected in the GC-FID chromatogram. Individual compounds were quantified as relative abundances, normalized to the internal standard (nonyl acetate) and expressed as relative peak area percentages. The mass spectra and retention indices (using alkanes C7–C30) of the compounds were compared with those in the NIST11 library and available synthetic standards for compound identification.

### Data Analyses

Host preference of the phloem-feeding insects was analyzed using generalized linear mixed models (GLMM) with a *Poisson* distribution, employing the *glmer* function from the *lme4* package, with time (3, 6, and 9 days) included as a fixed effect and plant pair included as a random effect to account for repeated measures. When overdispersion was detected, models were refitted using GLMM *negative binomial* distribution, employing *glmmTMB* function and family *nbinom2* from the *glmmTMB* package. Phloem-feeding insect performance was analyzed using generalized linear models (GLM) with a *Poisson* distribution, with model significance assessed by comparison to a null model. When overdispersion was present, data were reanalyzed using *quasi-Poisson* or *negative binomial* distribution, using the *glm.nb* function in the *MASS* package. Overdispersion was assessed using *check_overdispersion* function from the *performance* package.^78^ Model fit and significance were evaluated using AIC comparison. For ladybug prey preference assays, nonchoice test data were modeled using GLM with a *Poisson* distribution, while dual-choice assay data were analyzed using GLM with a *binomial distribution*. Choice data from the olfactometer assays were analyzed using GLMM with a *binomial* distribution, employing the *glmer* function from the *lme4* package, including plant pairs as a random factor to account for pseudoreplication. Volatile composition and the leaf secondary metabolite profiles were compared among treatments using multivariate approaches. Data were standardized using log-transformation and Pareto scaling.^79^ Differences in volatile composition among groups were analyzed using permutational multivariate analysis of variance (PERMANOVA), based on a Euclidean distance matrix (999 permutations) using the *adonis2* function from the *vegan* package. Permutational multivariate within-group dispersion analysis (PERMDISP) was performed using the *adonis* and *betadisper* function from the *vegan* package, with posthoc pairwise comparisons conducted via the *pairwise.adonis2* function from the *pairwiseAdonis* package. Principal component analysis (PCA) was performed using the *prcomp* function in the *stats* package. The phytohormone levels, individual volatile compounds, and leaf secondary metabolites were compared using ANOVA on log-transformed data, followed by Tukey’s Honest Significant Difference (HSD) test, using the *TukeyHSD* function in the *stats* package. All analyses were conducted using *R* software version 4.4.1,^80^ and package versions are provided in the Supporting Information (Table S1).

## Results

### Host Preference by Phloem-Feeding Insects

Prior colonization by either mealybugs or scales did not affect plant’s attractiveness to the other species. Mealybugs exhibited no preference for uninfested plants versus scale-infested plants (χ^2^ = 0.58, df = 1; *p* = 0.44, Figure 2A). Similarly, scales did not show preference between uninfested plants and those infested by mealybugs (χ^2^ = 0.65, df = 1; *p* = 0.42, Figure 2B). However, a significant effect of time (3, 6, and 9 days) was detected to mealybug choice (χ^2^ = 50.67, df = 2, *p* < 0.001), with no significant interaction between time and plant infestation treatment (χ^2^ = 1.96, df = 2, *p* = 0.37). Likewise, time significantly affected scale choice (χ^2^ = 32.50, df = 2, *p* < 0.001), with no significant interaction between time and plant infestation treatment (χ^2^ = 0.33, df = 2, *p* = 0.85), indicating temporal increase in the number of insects settling on plants, independent of plant infestation status (Figure 2).

**Figure 2:**
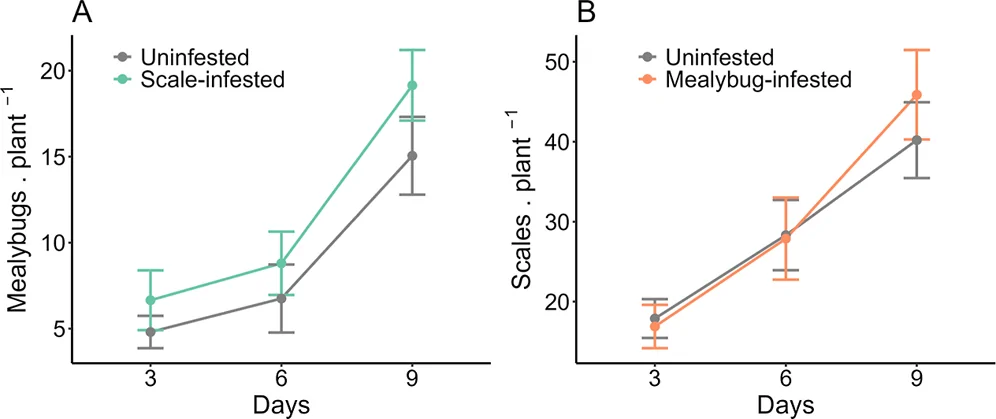
Host preference of phloem-feeding insects between *Coffea arabica* plants infested with nonconspecifics or uninfested plants in dual-choice assays over time. (A) Mealybugs (*Planococcus citri*) preference between uninfested and scale-infested plants (*n* = 20); (B) scales (*Coccus viridis*) preference between uninfested and mealybug-infested plants (*n* = 25). Dots represent the mean numbers ±SE of scales or mealybugs on the plant treatment at a time point.

### Phloem-Feeding Insects’ Performance

The performance of the two phloem-feeding insects on plants infested with nonconspecifics was asymmetrically affected. Mealybugs showed reduced performance when sharing the host plant with scales than when colonizing uninfested plants. There were approximately 22% more adults and 24% more mealybug nymphs on uninfested plants than on plants coinfested with scales (adults: χ^2^ = 6.33, df = 1.12; *p* = 0.01, nymphs: χ^2^ = 3.99, df = 1.22; *p* = 0.04, Figure 3A). On the other hand, the presence of mealybugs did not affect the performance of scales on coffee plants. The number of adult scales showed no significant difference on plants coinfested with mealybugs compared to uninfested plants (χ^2^ = 0.40, df = 1.22; *p* = 0.84, Figure 3B). Similarly, the number of scale nymphs did not differ between uninfested and mealybug-infested plants (χ^2^ = 3.33, df = 1.22; *p* = 0.07, Figure 3B).

**Figure 3:**
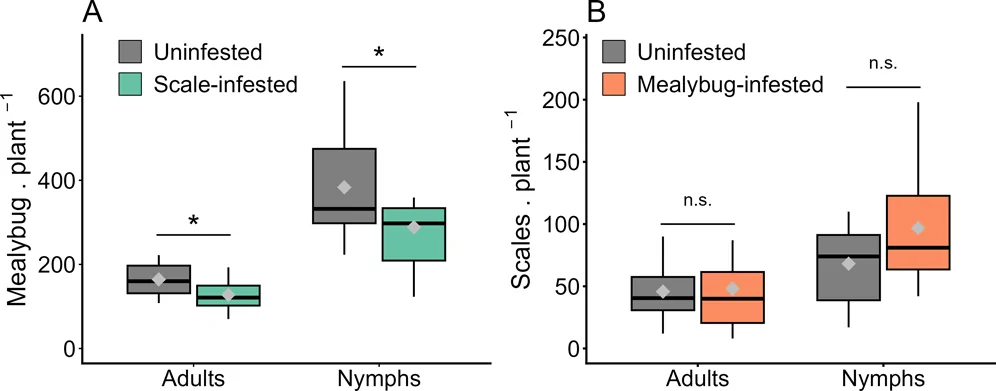
Performance of phloem-feeding insects in *Coffea arabica* plants infested with nonconspecifics or uninfested plants in no-choice assay. (A) Mealybugs (*Planococcus citri*) performance on plants previously infested with scales (scale-infested) (*n* = 12) or not (uninfested) (*n* = 12); (B) scales (*Coccus viridis*) performance on plants previously infested with mealybugs (mealybug-infested) (*n* = 12) or not (uninfested) (*n* = 12). Performance was evaluated based on the numbers of adults and nymphs found on coffee plants after 30 days of infestation. Boxes represent the interquartile range, whiskers indicate the minimum and maximum values, and the line within boxes represents the median. * indicates significant differences between single infested and uninfested plants according to GLM, negative binomial distribution (*p* < 0.05).

### Phytohormones

The jasmonates were differentially regulated by infestations of mealybugs and scales in coffee plants, and coinfestation by these phloem-feeding insects resulted in levels similar to those observed in mealybug-infested plants. Although the amounts of *cis*-(+)-12-oxophytodienoic acid (*cis*-OPDA), the precursor of jasmonates, were similar across all treatments (F_3,39_ = 1.33, *p* = 0.277, Figure 4A), the levels of JA and its derivatives JA-Ile, OH-JA, and COOH-JA-Ile were significantly higher in mealybug-infested plants and multiple-infested plants compared to uninfested and scale-infested plants (JA: F_3,39_ = 111.3, *p* < 0.001; JA-Ile: F_3,39_ = 37.47, *p* < 0.001; OH-JA: F_3,39_ = 62.91, *p* < 0.001; COOH-JA-Ile: F_3,39_ = 15.75, *p* < 0.001, Figure 4B–E and Table S2). Scale infestation, in turn, only affected OH-JA levels, which were downregulated compared to the other treatments (Figure 4D). SA levels were upregulated in plants infested either with scales or mealybugs compared to uninfested plants (F_3,39_ = 4.14, *p* = 0.012, Figure 4F), with no significant differences relative to multiple-infested plants. Scale infestation also reduced ABA levels compared to uninfested plants and the other infestation treatments (F_3,39_ = 18.14, *p* < 0.001, Figure 4G). Nevertheless, mealybug infestation alone or in coinfestation with scales did not alter ABA levels.

**Figure 4:**
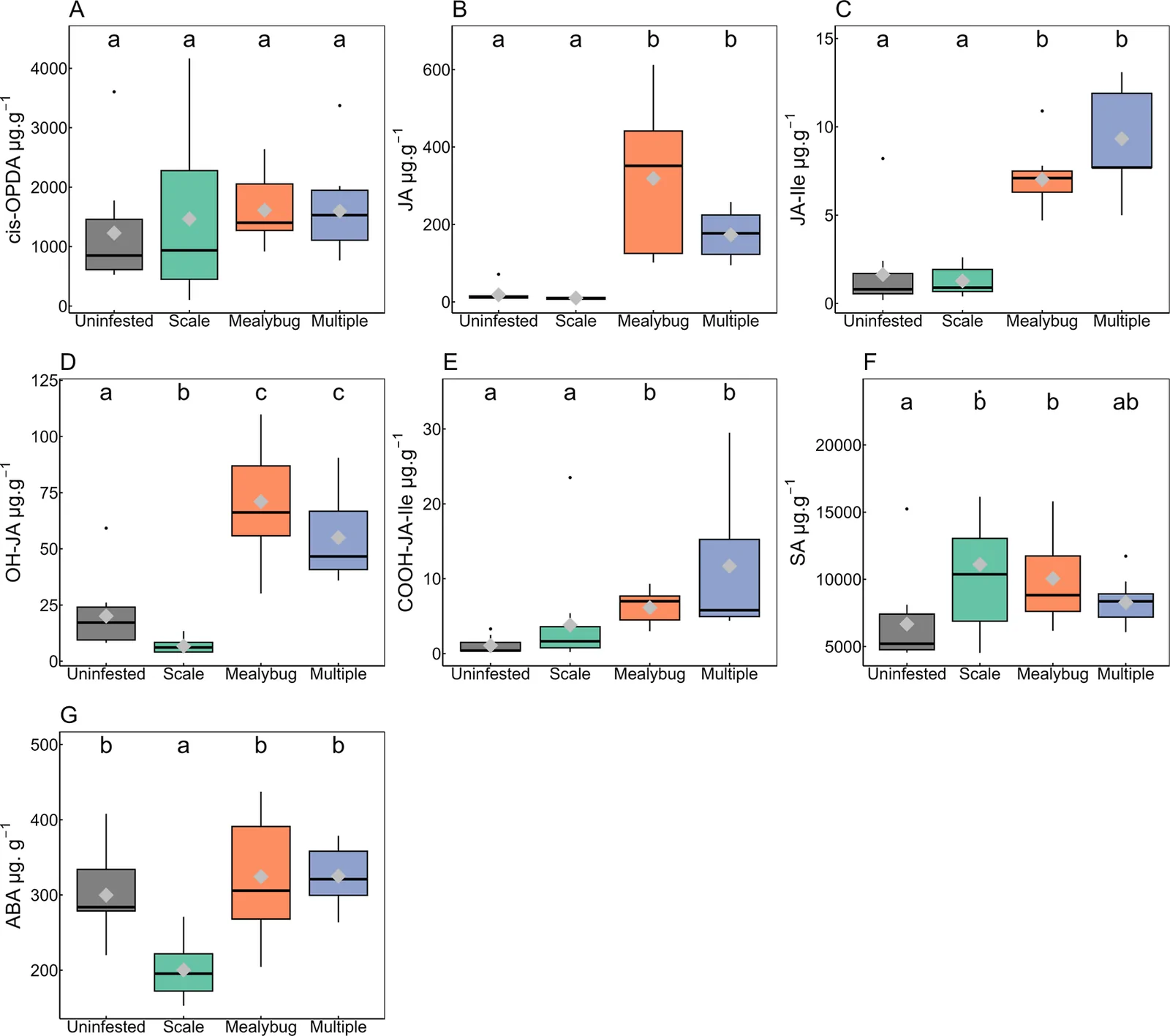
Levels of phytohormones (μg. g^–1^ dry leaf weight) in uninfested, scale-infested (*Coccus viridis*), mealybug-infested (*Planococcus citri*), and multiple-infested (scale + mealybug) *Coffea arabica* plants (*n* = 10–12). (A) *cis*-12-oxo-phytodienoic acid (OPDA); (B) jasmonic acid (JA); (C) sum of jasmonic acid isoleucine (JA-Ile); (D) hydroxylated jasmonic acid (OH-JA); (E) jasmonoyl-isoleucine (COOH-JA-Ile); (F) salicylic acid (SA); (G) Abscisic-acid (ABA). Boxes represent the interquartile range, whiskers indicate the minimum and maximum values, and the line within boxes represents the median. Different letters above bars indicate significant differences according to Tukey’s test (*p* < 0.05).

### Targeted Analysis of Secondary Metabolites

LC analysis of selected coffee secondary metabolites revealed differential induction by mealybug and scale infestation in coffee plants, and coinfestation resulted in metabolite levels similar to those observed in mealybug-infested plants. Infestation by mealybugs alone or in coinfestation with scales increased the amounts of caffeine (F_3,43_ = 53.23, *p* < 0.01, Figure 5A), catechin (F_3,43_ = 16.34, *p* < 0.01, Figure 5B), epicatechin (F_3,43_ = 14.27, *p* < 0.01, Figure 5C) and neochlorogenic acid (F_3,43_ = 19.02, *p* < 0.01, Figure 5E) compared to uninfested plants and those infested with scales alone (Figure 5 and Table S3). In contrast, infestation by scale-infested plants exclusively induced higher levels of the alkaloid theophylline, which was not detected in uninfested plants or the other infestation treatments (F_3,43_ = 15.55, *p* < 0.01 Figure 5D). Additionally, higher levels of tiliroside were detected in multiple-infested plants compared to uninfested and scale-infested plants, but they were similar to that in mealybug-infested plants (F_3,43_ = 4.95, *p* < 0.01 Figure 5F). Significant compositional differences were observed across all treatments (F_3,43_ = 17.01, R^2^ = 0.54, *p* < 0.01), with no significant variance within groups (F_3,43_ = 1.27, *p* = 0.29). However, metabolite composition of mealybug-infested and multiple-infested plants was similar (F_3,43_ = 2.55, R^2^ = 0.11, *p* = 0.12, Table S4).

**Figure 5:**
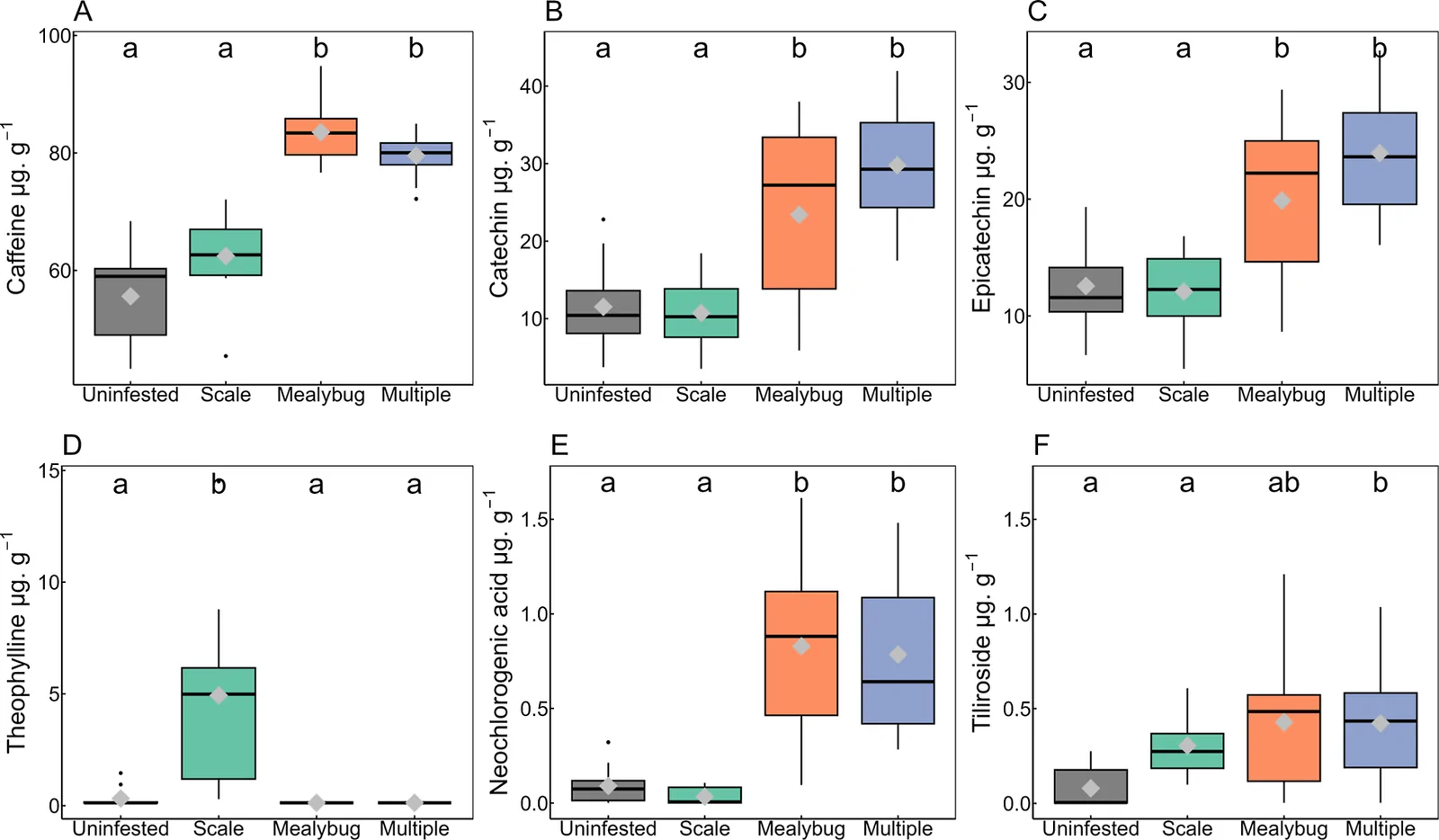
Amounts of selected secondary plant metabolites (μg g^–1^ dry leaf tissue) in uninfested, scale-infested (*Coccus viridis*), mealybug-infested (*Planococcus citri*), and multiple-infested (mealybug + scale) *Coffea arabica* plants (*n* = 10–12). (A) Caffeine; (B) catechin; (C) epicatechin; (D) theophylline; (E) neochlorogenic acid; (F) tiliroside. Boxes represent the interquartile range, whiskers indicate the minimum and maximum values, and the line within boxes represents the median. Different letters above bars indicate significant differences according to Tukey’s test (*p* < 0.05).

### Prey Preference of *Cr. montrouzieri*

The ladybug *Cr. montrouzieri* consumed similar numbers of adult mealybugs and scales when provided separately (nonchoice assay) (χ^2^ = 1.18, df = 1.38; *p* = 0.28, Figure 6A). However, in the dual-choice assay, where ladybugs could choose between the two prey species, they showed a preference for consuming mealybugs (χ^2^ = 4.00, df = 1.38; *p* = 0.04, Figure 6B). The mean consumption was 23.8 ± 1.98 (mean ± SE) mealybugs compared to 9.6 ± 1.7 (mean ± SE) scales, with ladybugs consuming 2.4 times more mealybugs than scales.

**Figure 6:**
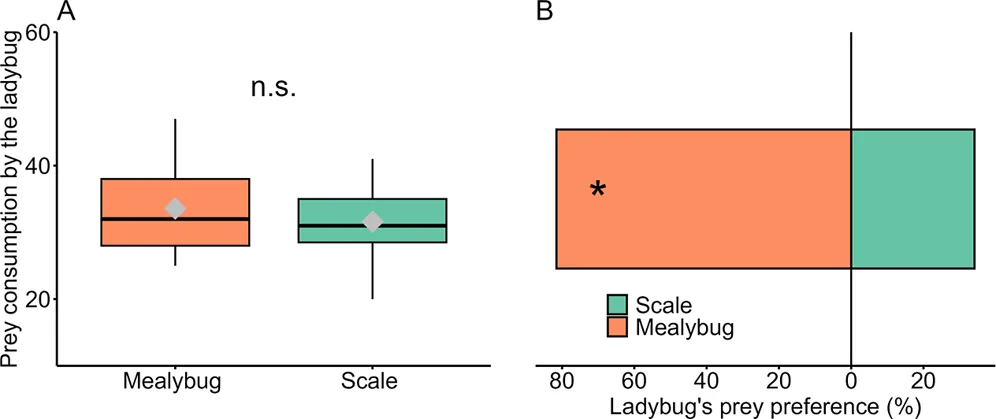
Prey preference of *Cryptolaemus montrouzieri* females for *Planococcus citri* (mealybug) (*n* = 17) and *Coccus viridis* (scale) (*n* = 23) in (A) a nonchoice assay and (B) a dual-choice assay (*n* = 20). “n.s.” indicates no statistically significant difference between treatments. * indicates significant differences between scale and mealybug-infested plants according to GLM, binomial distribution (*p* < 0.05).

### Olfactory Preference of *Cr. montrouzieri* to Plant Volatiles

In olfactometer assays, the ladybugs *Cr. montrouzieri* were more attracted to volatiles from plants infested with either *P. citri* (mealybug) or *Co. viridis* (scale) compared to those from uninfested plants (mealybug: χ^2^ = 8.88, df = 1.95; *p* < 0.01; scales: χ^2^ = 6.62, df = 1113; *p* = 0.01, Figure 7). However, when exposed to volatiles from mealybug-infested and scale-infested plants, the ladybugs did not differentiate between the two treatments (χ^2^ = 0.15, df = 1101; *p* = 0.70, Figure 7). Volatiles from multiple-infested plants were less attractive to *Cr. montrouzieri* than those from mealybug-infested plants (χ^2^ = 3.89, df = 1.79; *p* = 0.04, Figure 7). However, the ladybugs did not differentiate between volatile emissions from scale-infested and multiple-infested plants (χ^2^ = 1.84, df = 1103; *p* = 0.18, Figure 7). Similarly, *Cr. montrouzieri* showed no preference between volatiles from uninfested and multiple-infested plants (χ^2^ = 0.08, df = 1107; *p* = 0.85, Figure 7).

**Figure 7:**
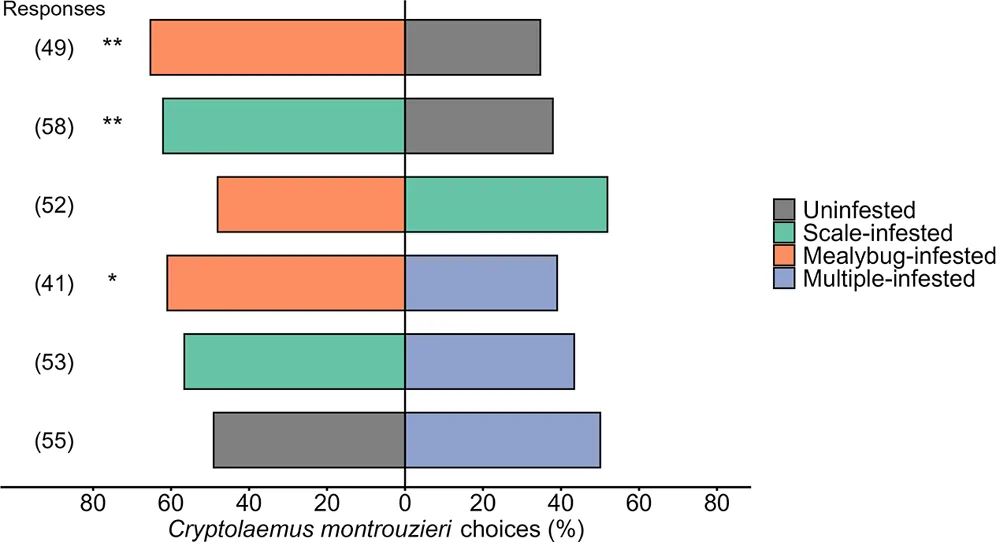
Olfactory preference of the predatory ladybug *Cryptolaemus montrouzieri* in two-choice olfactometer assays for volatiles emitted from uninfested, scale-infested (*Coccus viridis*), mealybug-infested (*Planococcus citri*), and multiple-infested (mealybug + scale) *Coffea arabica* plants (*n* = 6 plant pairs for each combination). *p < 0.05; **p < 0.01 according to GLM, binomial distribution (*p* < 0.05). Numbers in brackets indicate the number of insects responding in the assay.

### Plant Volatiles

A total of nine VOCs were identified in the headspace of coffee plants under different treatments, comprising two terpenes, three benzenoids, two alkanes, and two unidentified compounds (Table 1). Significant differences in volatile compositions were observed among treatments (F_3,18_ = 8.10, R^2^ = 0.57, *p* < 0.01), with no significant differences in multivariate dispersion among treatments (F_3,18_ = 0.65, *p* = 0.61). The PCA clearly separated the volatile blend of uninfested plants from those of the infestation treatments along PC1 and PC2, while the volatile blend of multiple-infested plants partially separated from those of mealybug-infested or scale-infested plants (Figure 8 and Table S5).

**Table 1:** Volatile Organic Compounds Detected in the Headspace of Coffee Plants (*Coffea arabica*) Subjected to Different Treatments: Uninfested, Scale Infested (*Coccus viridis*), Mealybug-Infested (*Planococcus citri*), and Multiple-Infested (Mealybug + Scale) Plants (*n* = 5–6 Plants)^a^

compounds	RI	uninfested	scale-infested	mealybug-infested	multiple-infested	F	*p*
***terpenes***		0.31 ± 0.05c	7.47 ± 2.70a	0.50 ± 0.28bc	6.07 ± 2.68ab	7.21	**<0.01**
**DMNT** ^b^	1106	0.27 ± 0.04b	3.94 ± 1.38a	0.24 ± 0.06b	4.80 ± 2.12a	3.50	**0.04**
**TMTT** ^c^	1572	0.04 ± 0.02b	3.52 ± 1.36b	0.26 ± 0.23bc	1.48 ± 0.50ac	8.75	**<0.01**
***benzenoids***		1.26 ± 0.16c	4.29 ± 0.64ab	1.93 ± 0.25bc	5.59 ± 1.87a	11.05	**<0.01**
**methyl salicylate** ^d^	1170	1.26 ± 0.16	4.07 ± 0.62	1.22 ± 0.26	4.95 ± 1.69	3.47	0.07
**ethyl benzoate** ^d^	1147	0.00 ± 0.00b	0.13 ± 0.02a	0.01 ± 0.00b	0.23 ± 0.09a	11.44	**<0.01**
**1-octen-3-ol** ^d^	967	0.00 ± 0.00a	0.10 ± 0.05ab	0.70 ± 0.22c	0.41 ± 0.17bc	11.15	**<0.01**
***alkanes***		1.08 ± 0.14	1.05 ± 0.09	0.76 ± 0.07	0.85 ± 0.17	1.62	0.22
**5-isobutyl-nonane**	1097	0.08 ± 0.01	0.07 ± 0.00	0.05 ± 0.00	0.06 ± 0.01	1.91	0.16
**3,** **7-dimethyldecane**	1067	1.01 ± 0.13	0.97 ± 0.09	0.71 ± 0.07	0.80 ± 0.16	1.27	0.31
***Unknown compounds***							
**unknown 1**	1117	0.10 ± 0.02	0.06 ± 0.01	0.05 ± 0.01	0.05 ± 0.00	3.09	0.06
**unknown 2**	1265	0.00 ± 0.00b	0.00 ± 0.00b	0.29 ± 0.13a	0.13 ± 0.04a	11.79	**<0.01**
***total production***		2.76 ± 0.37b	12.86 ± 3.30a	3.52 ± 0.34ab	12.7 ± 4.69a	7.08	**<0.01**

a Values are expressed as mean relative amount ±SE (ng. g dry tissue^–1^). Lowercase letters indicate statistically significant differences between treatments according to Tukey’s test (*p* < 0.05).

b DMNT: (*E*)-4,8-dimethyl-1,3,7-nonatriene.

c TMTT: (*E,E*)-4,8,12-trimethyl-1.3,7,11-tridecatetraene.

d Compound identification was verified by retention times and mass spectra of synthetic standards.

**Figure 8:**
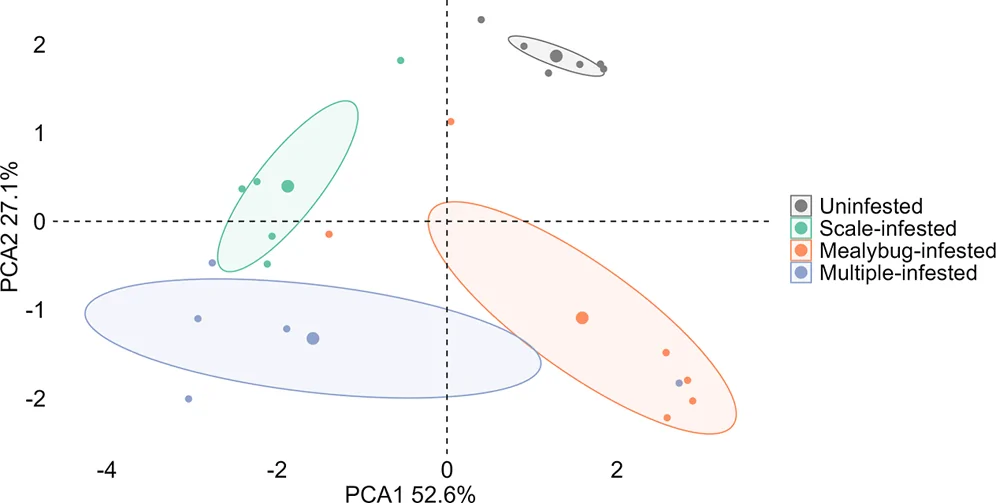
Principal component analysis (PCA) of the relative amounts of volatile compounds in the blend emitted from coffee plants (*Coffea arabica*) under different treatments: uninfested, scale-infested (*Coccus viridis*), mealybug-infested (*Planococcus citri*), and multiple-infested (mealybug + scale) plants. Larger dots represent the treatment centroids and smaller dots represent individual samples (*n* = 5–6 plants). See Table 1 for the complete list of compounds.

Univariate analyses revealed that scale infestation, either alone or in combination with mealybugs on coffee plants, resulted in higher total amounts of volatile emissions compared with uninfested plants. In contrast, mealybug infestation alone induced intermediate total volatile amounts, which did not significantly differ from either uninfested plants or the other infestation treatments (Table 1). In terms of individual compounds, scale infestation elicited higher emissions of the terpenes (*E*)-4,8-dimethyl-1,3,7-nonatriene (DMNT) and (*E,E*)-4,8,12-trimethyl-1,3,7,11-tridecatetraene (TMTT) compared with both uninfested and mealybug-infested plants (Table 1). Multiple-infested plants released DMNT at levels similar to those emitted by scale-infested plants. Additionally, ethyl benzoate and unknown compound **2** were detected exclusively in the volatile blends of scale-infested and multiple-infested plants. Mealybug infestation alone resulted in higher emissions of 3,7-dimethyldecane compared with uninfested plants. Furthermore, the volatile blend emitted by mealybug-infested plants contained higher levels of the fungal-associated compound **1**-octen-3-ol than those from uninfested and scale-infested plants, but levels similar to those emitted from multiple-infested plants. When comparing total amounts by chemical group, scale infestation, either alone or in combination with mealybugs, induced greater emissions of terpenes and benzenoids compared with uninfested plants (Table 1). Conversely, mealybug infestation alone elicited terpene and benzenoid emissions similar to those of uninfested plants and intermediate between the levels observed in scale-infested and multiple-infested plants. In contrast, the total alkane emissions did not differ significantly among treatments.

## Discussion

### Interactions between the Scale and Mealybug in Coffee Plants

Our study revealed that colonization by either scales or mealybugs did not influence the subsequent arrival of the nonconspecific phloem-feeding insect. However, while mealybug infestation did not affect scale performance, scale infestation had a negative effect on mealybug performance. Together, these results refute our hypothesis that colonization by phloem-feeding insects would positively affect the colonization and performance of the nonconspecific insect.

Examination of plant chemical changes associated with the interactions among scales, mealybugs, and coffee plants revealed that infestation by either phloem-feeding insect led to the accumulation of SA, an expected plant response to herbivory of the phloem-feeding guild.^6,13^ However, single infestations elicited distinct regulatory patterns in the jasmonate and ABA signaling pathways. In scale-infested plants, SA accumulation was associated with the suppression of ABA and the inactive JA form OH-JA, while levels of the active JA form JA-Ile remained unchanged. The suppression of ABA observed in scale-infested plants may result from activation of SA, as their pathways can interact antagonistically.^81,82^ Similarly, the reduced accumulation of OH-JA suggests constrained jasmonate accumulation, consistent with the antagonistic interaction between the SA and JA pathways,^16^ although time-course hormonal profiling, marker-gene expression analysis, and experimental manipulation would be needed to confirm this regulatory interaction. In contrast, mealybug feeding was associated with the activation of both SA and JA signaling pathways, consistent with a previous report from a shorter-term mealybug infestation in coffee plants.^34^ Interestingly, the hormonal profile in scale-infested plants did not facilitate the arrival or establishment of the mealybug. Although such suppression of jasmonates has been reported to enhance herbivore performance in other systems,^19,20,83,84^ our findings align with evidence showing that increased salicylate signaling induced by one phloem-feeding herbivore can negatively affect the performance of another herbivore within the same feeding guild.^17,28–30,85^

First-instar nymphs of both scales and mealybugs actively search for and select feeding sites before establishing colonies,^56,58,59,68^ whereas adult females are far less mobile or even stationary.^56,58^ Consequently, the ability of first-instar nymphs to identify suitable or high-quality hosts is critical for colony establishment. In this context, plant volatile emissions have been shown to orient nymphs of several species within the superfamily Coccoidea during host selection.^86–88^ In our study, infestation by either scales or mealybugs induced distinct profiles of volatile and nonvolatile secondary metabolites in coffee plants, supporting our hypothesis that the regulation of herbivore-induced plant defenses is species-specific. Notably, for mealybugs, the chemical changes elicited by scale infestation did not function as reliable cues of host quality, as mealybugs did not discriminate between scale-infested plants and uninfested plants, despite exhibiting poorer performance on the former.

Mealybug infestation elicited a more pronounced chemical response in coffee plants, increasing the levels of most of the assessed secondary metabolites compared with scale infestation. This pattern co-occurred with the elevation of both SA and JA, including its derivatives such as JA-Ile, OH-JA, and COOH-JA-Ile. Among the metabolites upregulated by mealybug infestation, caffeine and chlorogenic acid have been reported to act as deterrents to the scale, inducing increased locomotor activity and reduced feeding.^56,70^ Similarly, catechin and epicatechin have shown strong deterrent effects against a range of herbivores.^89–92^ Tiliroside, which was discretely induced by mealybug infestation, is known to function as an oviposition deterrent for the coffee leaf miner *Leucoptera coffeella*.^93^ Despite the upregulation of these putative defensive compounds, scale performance was not affected under coinfestation with mealybugs, indicating that scales are able to tolerate or cope with these chemical defenses in coffee plants.

In contrast, scale infestation induced an increase only in the alkaloid theophylline among the secondary metabolites assessed, a pattern that coincided with the exclusive activation of the salicylate signaling pathway. Like caffeine, theophylline is a methylxanthine, but it serves as a precursor in caffeine biosynthesis and represents a less stable form.^94–96^ Although studies addressing the role of theophylline as an antiherbivore defense compound are scarce, alkaloids are broadly recognized for their defensive properties, exhibiting species-specific effects on herbivores.^97,98^ Notably, among the assessed secondary metabolites, the accumulation of theophylline in scale-infested plants coincided with reduced mealybug performance. This pattern highlights the need for future assays testing the potential deterrent or toxic effects of theophylline on mealybugs to confirm its role in reducing their performance. Moreover, the metabolites quantified here represent only a subset of the plant’s full chemical phenotype, and broader untargeted metabolomic approaches are needed to further investigate the metabolites shaping these interactions.

Contrary to our hypothesis that multiple infestation by phloem-feeding insects would synergistically increase herbivore-induced defenses compared with single infestations, the levels of nonvolatile compounds in multiple-infested plants were similar to those observed in mealybug-infested plants. Mealybugs were numerically more abundant both in single infestation or under coinfestation with scales, likely reflecting their shorter life cycle (about 40 to 52 days) compared with scales (about 50 to 70 days).^56,57,68^ Consistently, multiple-infested plants exhibited higher mealybug abundance, with 85% of adults and 78% of nymphs being mealybugs, highlighting the dominance of this species in the infestation. The chemical profile of multiple infested plants more closely resembling that of mealybug-infested plants may therefore reflect, at least in part, the dominance of mealybugs on the plants relative to scales under coinfestation conditions, a pattern that is consistent with evidence that defense responses can be modulated by the most prevalent and potentially damaging herbivore.^31,99^

### Interactions among Scales, Mealybugs and the Predator

The predatory ladybug *Cr. montrouzieri* has been widely introduced as a biocontrol agent against Pseudococcidae, primarily targeting the mealybug.^73,74^ However, it is also effective in controlling other hemipteran species, including the scale.^72,100,101^ In our study, *Cr. montrouzieri* females consumed mealybugs and scales at similar rates when offered separately, but preferred mealybugs when given a choice. Although ladybugs were reared exclusively on mealybugs prior to the assays, the equivalent consumption of scales in the nonchoice experiment indicates that prior diet did not impair predation on the alternative prey. Moreover, a previous study showed that laboratory conditioning of *Cr. montrouzieri* on other coccoid species over several generations did not alter its preference for *P. citri*.^102^ Thus, the preference for *P. citri* observed under choice conditions may reflect the well-established role of *Cr. montrouzieri* as a specialist predator of Pseudococcidae,^74^ rather than a response driven solely by rearing diet.

The preference for mealybugs over scales likely involves prey-related traits beyond olfactory cues. Although *Cr. montrouzieri* exploits prey chemical cues at short distances, it does not differentiate its preferred prey (*P. citri*) from alternative coccoids based on them.^102^ Mealybugs produce structurally complex wax filaments that can provide tactile linked to thigmotactic behavior and increased egg production in *Cr. montrouzieri*.^103,104^ In addition, although the ability of *Cr. montrouzieri* to use visual cues in prey location remains unknown, other coccinellid predators exploit visual cues, such as prey color and shape, which may contribute to close-range prey discrimination or the detection of prey-rich patches.^105^

Contrary to our hypothesis, the preference of female ladybugs for mealybugs observed in the prey-choice assay did not translate into an olfactory preference for volatile emissions from plants infested with either scales or mealybugs, as *Cr. montrouzieri* was equally attracted to them. Thus, HIPV emissions from singly infested plants represent reliable signal of prey presence to *Cr. montrouzieri* but do not convey specific information about prey identity or preference. This dissociation, together with evidence that *Cr. montrouzieri* does not differentiate its preferred prey from alternative coccoids based on contact chemical cues alone,^102^ suggests that prey selection may rely more on tactile and visual signals than on olfactory information, whether at long or short-range.

Although the ladybug did not discriminate between the volatile blends emitted from mealybug-infested and scale-infested plants, the composition of these blends differed primarily in quantitative terms. Only one compound (unknown 2) was exclusive to mealybug-infested plants, whereas ethyl benzoate was detected in scale-infested plants, which also emitted higher amounts of several compounds, including terpenes and benzenoids. As predator attraction to plant volatile blends is often mediated by the relative ratios of key compounds within the blend,^39,47,106^ similar proportional relationships among VOCs may explain why blends from mealybug- and scale-infested plants were equally attractive to *Cr. montrouzieri*. While no study has specifically tested the activity of isolated plant volatile compounds in behavior or antennal responses of *Cr. montrouzieri*, some volatiles detected in the headspace of coffee plants are known as attractants for other ladybugs, such as DMNT^107,108^ and methyl salicylate (MeSA).^109,110^ Further research is therefore required to elucidate the biological activity of individual compounds and their mixture in mediating the responses of *Cr. montrouzieri*.

The recognition and attractiveness of volatile emissions from plants infested by a single prey species to natural enemies are well-documented.^38,40,111,112^ However, in natural settings, plants are attacked by a community of herbivores, which can interfere with the chemical communication among plants, herbivores, and natural enemies.^22^ We hypothesized that plants coinfested by mealybugs and scales, both prey species, would not alter the attractiveness of HIPVs relative to singly infested plants. Contrary to this expectation, *Cr. montrouzieri* was not attracted to volatile emissions from multiple-infested plants compared with uninfested plants, indicating a disruption of chemically mediated tritrophic interaction. When given a choice between odors from mealybug-infested and multiple-infested plants, *Cr. montrouzieri* preferentially oriented toward mealybug-infested plants, despite the lower number of prey individuals on these plants compared to multiple-infested plants, which harbored both mealybugs and scales. Because scale-infested plants emitted volatiles that attracted the ladybug, we expected a similar outcome when comparing scale-infested plants and multiple-infested plants. However, *Cr. montrouzieri* did not discriminate between these two treatments. In this specific olfactory context, the ladybug was either equally attracted to both treatments or was unable to perceive the volatile blend emitted by scale-infested plants as attractive. Supporting this interpretation, volatile analyses revealed that the blend emitted by multiple-infested plants more closely resembled that of scale-infested plants than that of mealybug-infested plants. For example, amounts of DMNT, TMTT, and ethyl benzoate were present in higher amounts in both scale- and multiple-infested plants than in other treatments. Although not statistically significant, the total amount of MeSA was approximately 3-fold higher in the blend emitted from scale-infested and multiple-infested plants than in mealybug-infested plants. MeSA is well-known attractant of natural enemies,^113,114^ including ladybugs^115,116^ although its effectiveness can be concentration-dependent and inconsistent in some cases.^110,115–117^ Even though the current data set does not allow identification of which specific compounds or blend ratios are biologically relevant to *Cr. montrouzieri*, changes in the relative ratios among key volatile compounds within the blend may have contributed to the predator’s behavioral response under multiple infestation.

Notably, the volatile emission of multiple-infested plants also contained some compounds at similar amounts to those detected in mealybug-infested plants, such as 1-octen-3-ol. This compound is derived from fungal oxidation of linolenic acid and has been associated with microbial communities colonizing hemipteran honeydew.^118,119^ Both scales and mealybugs produce honeydew as a byproduct of phloem feeding, but its composition appears to be species-specific, potentially supporting distinct microbial communities.^120^ Honeydew-associated microorganisms have recently been shown to influence plant defense signaling,^121^ suggesting that microbial activity on honeydew may extend beyond volatile production to shape broader plant responses. Volatiles produced by honeydew-associated microorganisms have been reported to function as foraging cues for natural enemies, including coccinellids.^118,122–124^ Feeding on honeydew has also been shown to enhance predator mobility and prey consumption,^124^ suggesting that 1-octen-3-ol may signal a carbohydrate source occasionally exploited by *Cr. montrouzieri*.^65,123^ It is therefore plausible that differences in honeydew composition and associated microbial activity between mealybugs and scales contributed to shaping the volatile blend perceived by *Cr. montrouzieri*. Whether honeydew-derived microbial volatiles play a meaningful role in mediating predator attraction in this system, however, remains to be directly investigated.

Despite the presence of several potentially attractive compounds in the volatile blend of multiple-infested plants, overlapping with those emitted from singly infested plants, the ladybug did not discriminate between this odor source and the uninfested plant. The lack of attractiveness may result from a specific combination of compounds or from altered ratios that are not recognized by the ladybug as a reliable cue of prey presence. Among the compounds detected in coffee plant headspace, only ethyl benzoate, which was present in both scale- and multiple-infested plants, has been reported to exhibit repellent or insecticidal properties against insects.^34,125,126^ Further studies are therefore needed to elucidate the mechanism underlying the lack of attractiveness of the volatile blends emitted by plants coinfested with both prey species to *Cr. montrouzieri*, using behavioral assays and olfactory or electroantennographic approaches with individual compounds and mixtures.

Our results emphasize the specificity of plant defenses in multitrophic systems, particularly among phloem-feeding herbivores, which are often assumed to share similar hormonal mechanisms. Despite belonging to the same feeding guild and being closely related, a single infestation by either the scale *Co. viridis* or mealybug *P. citri* triggered distinct phytohormonal responses. These infestations did not affect the likelihood of subsequent colonization by a nonconspecific insect but resulted in an asymmetric plant-mediated resistance against the mealybug, while promoting a similar recruitment of *Cr. montrouzieri* through volatile emissions. Moreover, coinfestation by mealybug and scale did not amplify herbivore-induced plant defenses instead inducing similar chemical profiles as those observed under single infestations. Despite emitting a volatile blend resembling that of scale-infested plants, multiple infestation disrupted the volatile-mediated attraction of *Cr. montrouzieri* to coffee plants. However, because this study captured a single time point (30 days postinfestation), time-course analyses are needed to assess the temporal dynamics of these defense responses. From an applied perspective, our results indicate that coinfestation by the two pests in coffee plantations may negatively impact the recruitment and prey location efficiency of *Cr. montrouzieri*, potentially compromising its effectiveness as a biological control agent. Further research is required to identify whether specific VOCs or their mixtures influence the behavior of *Cr. montrouzieri* and how these blends could be optimized to improve the attraction of natural enemies under complex infestation scenarios.

## Supplementary Material



## Data Availability

Data sets generated in this study are available from the OSF data repository (DOI: 10.17605/OSF.IO/XD8GS ).

## Notes

R packages and versions used in the data analyses (Table S1); levels of phytohormones in leaves of infested plants (Table S2); amounts of secondary plant metabolites in leaves of infested plants (Table S3); PERMANOVA, PERMUTEST, and pairwise tests of the relative amounts of secondary plant metabolites (Table S4); PERMANOVA, PERMUTEST, and pairwise tests of the relative amounts of volatile organic compounds (Table S5) (PDF)

## Abbreviations

VOCs: volatile organic compounds
HIPVs: herbivore-induced plant volatiles
JA: jasmonic acid
JA-Ile: jasmonic acid isoleucine
OH-JA: hydroxylated jasmonic acid
COOH-JA-Ile: jasmonoyl-isoleucine
SA: salicylic acid
ABA: abscisic acid
DMNT: (E)-4,8-dimethyl-1,3,7-nonatriene
TMTT: (E,E)-4,8,12–535 trimethyl-1.3,7,11-tridecatetraene.
