# Supplementary material for: Single and Multiple Infestation by Coccoid Pests in Coffee Shape Plant Chemical Defenses and Interactions Across Trophic Levels
Source: J Agric Food Chem. 2026 Aug 8;74(33):25960–75. doi: 10.1021/acs.jafc.6c04549 (PMC13557442; doi:10.1021/acs.jafc.6c04549)
Supplement: Supplementary file 1 [file jf6c04549_si_001.pdf]

Single and multiple infestation by coccoid pests in coffee shape plant chemical defenses and interactions across trophic levels

Enggel Beatriz Silva Carmo[1*], Renato C. Macedo-Rego[3], Alexander N. Borg[2], John C. Caulfield[2], Bruna Corrêa-Silva[1], Axel Mithöfer[4], Arodí P. Favaris[5], José Mauricio Simões Bento[5], Maria Fernanda Gomes Villalba Peñaflor[1*]

[1]Departamento de Entomologia, Universidade Federal de Lavras (UFLA), Lavras 37203-202, Brazil

[2]Translating Biotic Interactions, Rothamsted Research, Harpenden AL5 2JQ, UK

[3]LAGE do Departamento de Ecologia, Instituto de Biociências, Universidade de São Paulo, São Paulo 05508-090, Brazil

[4]Research Group Plant Defense Physiology, Max Planck Institute for Chemical Ecology, Jena D-07745, Germany

[5]Departamento de Entomologia e Acarologia, Escola Superior de Agricultura Luiz de Queiroz, Universidade de São Paulo, Piracicaba 13418-900, Brazil

*Corresponding Author: fernanda.penaflor@ufla.br, enggelcarmo@gmail.com

Table of Contents

Table S1 R packages and versions used in the data analyses S-2

Table S2 Levels of phytohormones in leaves of infested plants.......................................S-3

Table S3 Amounts of secondary plant metabolites in leaves of infested plants..............S-3

Table S4 PERMANOVA, PERMUTEST, and pairwise tests of the relative amounts of secondary plant metabolites S-4

Table S5 PERMANOVA, PERMUTEST, and pairwise tests of the relative amounts of volatile organic compounds S-4

Table S1. R packages and versions used in the data analyses conducted using *R* software (version 4.4.1).

PackageS	Version
**glmmTMB**	1.1.10
**lme4**	1.1-35.5
**pairwiseAdonis**	0.4.1
**performance**	0.12.3
**vegan**	2.6-8
**MASS**	7.3-60.2
**stats**	4.4.1

Table S2. Levels (mean ±SE µg. g^{-1} dry tissue) of phytohormones in leaves of uninfested, scale-infested (*Coccus viridis*), mealybug-infested (*Planococcus citri*), and multiple-infested (mealybug + scale) infested *Coffea arabica* plants.

**Treatments**	**Uninfested**	**Scale-infested**	**Mealybug-infested**	**Multiple-infested**	**F**	***P***
**SA**	6674.00±946.00b	11110.00±1571.00a	10053.00±1060.00ab	8293.00±484.00ab	4.14	**0.012**
**JA**	18.20±5.52a	9.98±1.02a	319.00±59.80b	173.00±18.00b	111.3	**<0.001**
**ABA**	300.00±16.20a	200.00±11.40a	324.00±25.00b	325.00±12.00b	18.14	**<0.001**
**JA-Ile**	1.64±0.69	1.27±0.22	7.02±0.61	9.33±0.81	37.47	**<0.001**
**cis-OPDA**	1228.00±271.00	1466.00±380.00	1613.00±208.00	1598.00±219.00	1.33	0.277
**OH-JA**	20.10±4.40a	6.71±0.86b	71.00±8.60ac	55.00±5.88ac	62.91	**<0.001**
**COOH-JA-Ile**	1.08±0.32a	3.82±1.86b	6.16±0.73c	11.70±2.94c	15.75	**<0.001**

SA- Salicilic acid, JA- Jasmonic acid, ABA – Abscisic acid, JA-Ile – Jasmonic acid isoleucine, cis-OPDA - cis-12-oxo-phytodienoic acid (OPDA); OH-JA hydroxylated jasmonic acid, COOH-JA-Ile jasmonoyl-isoleucine.

Table S3. Amounts (mean ±SE µg. g^{-1} dry tissue) of secondary plant metabolites in leaves of: uninfested, scale-infested (*Coccus viridis*), mealybug-infested (*Planococcus citri*), and multiple-infested (mealybug + scale) infested *Coffea arabica* plants.

**Treatments**	**Uninfested**	**Scale-infested**	**Mealybug-infested**	**Multiple-infested**	**F**	***P***
**Theophylline**	4.93±1.18b	0.22±0.14a	00.00±00.00b	00.00±00.00b	15.55	**<0.01**
**Caffeine**	62.50±2.02a	55.60±2.30a	83.50±1.58b	79.50±1.17b	53.23	**<0.01**
**Catechin**	10.72±1.27a	11.53±1.58a	23.42±3.27b	29.80±2.48b	16.34	**<0.01**
**Epicatechin**	12.1±0.99a	12.6±1.10a	19.88±2.06b	23.94±1.69b	14.27	**<0.01**
**Tiliroside**	0.31±0.05b	0.08±0.03ab	0.43±0.10a	0.42±0.10a	4.95	**<0.01**
**Neochlorogenic acid**	0.03±0.01b	0.10±0.03b	0.83±0.15a	0.79±0.13a	19.02	**<0.01**

Table S4. PERMANOVA, PERMUTEST, and pairwise tests of the relative amounts of of secondary plant metabolites found in coffee plants, *Coffea arabica*, across different treatments: uninfested, scale-infested (*Coccus viridis*), mealybug-infested (*Planococcus minor*), and multiple-infested (mealybug + scale) plants.

**Pairwise test**	**F**	**R^2**	***P***
**Mealybugs vs. Uninfested**	39.9	0.63	**<0.01**
**Scales vs. Uninfested**	4.12	0.16	**0.02**
**Scales vs. Mealybugs**	27.11	0.55	**<0.01**
**Mealybugs vs. Multiple infested**	2.55	0.11	0.12
**Scales vs. Multiple infested**	44.82	0.69	**<0.01**
**Uninfested vs. Multiple infested**	53.53	0.72	**<0.01**
**PERMANOVA**	17.01	0.54	**<0.01**
**PERMUTEST**	1.27		0.29

Table S5. PERMANOVA, PERMUTEST, and pairwise tests of the relative amounts of volatile organic compounds found in coffee plants, *Coffea arabica*, across different treatments: uninfested, scale-infested (*Coccus viridis*), mealybug-infested (*Planococcus minor*), and multiple-infested (mealybug + scale) plants.

**Pairwise test**	**F**	**R^2**	***P***
**Mealybugs vs. Uninfested**	5.92	0.37	**0.01**
**Scales vs. Uninfested**	24.52	24.52	**<0.01**
**Scales vs. Mealybugs**	13.39	13.39	**0.01**
**Mealybugs vs. Multiple infested**	3.68	0.29	0.06
**Scales vs. Multiple infested**	2.78	2.79	**0.04**
**Uninfested vs. Multiple infested**	10.21	10.21	**<0.01**
**PERMANOVA**	8.09	0.57	**<0.01**
**PERMUTEST**	0.65		0.61
